# Autophagy–Circulating Tumor DNA Axis in Molecular Cancer Research: Emerging Mechanisms, Therapeutic Targeting, and Translational Opportunities

**DOI:** 10.3390/ijms27104596

**Published:** 2026-05-20

**Authors:** Abdel Halim Harrath, Maroua Jalouli, Md Ataur Rahman

**Affiliations:** 1 Zoology Department, College of Science, King Saud University, Riyadh 11451, Saudi Arabia; hharrath@ksu.edu.sa; 2 Department of Biology, College of Science, Imam Mohammad Ibn Saud Islamic University (IMSIU), Riyadh 11623, Saudi Arabia; mejalouli@imamu.edu.sa; 3 Department of Oncology, Karmanos Cancer Institute, Wayne State University, Detroit, MI 48201, USA

**Keywords:** autophagy, circulating tumor DNA, liquid biopsy, cancer progression, therapeutic resistance, translational oncology

## Abstract

Autophagy is a self-degradative homeostatic mechanism that plays an important role in tumor viability, metabolic reprogramming, and drug resistance. Circulating tumor DNA (ctDNA) is fragmented DNA that comes from dying tumor cells and leaks out into the blood stream. ctDNA can now be measured through blood tests and is a non-invasive way to identify cancer. ctDNA has shown promise for early detection of cancer, prognosis, and monitoring treatment response in real time. There is emerging mechanistic evidence suggesting a potential relationship between autophagy and ctDNA dynamics which has been discussed as a new autophagy–ctDNA axis. Autophagy can affect ctDNA levels by promoting or suppressing apoptosis and necrosis of tumor cells. When autophagy is cytoprotective, less DNA would be shed into the bloodstream. When autophagy is inhibited or defective, more DNA would be released because of increased genomic instability. Stressors found within the tumor microenvironment (TME) like hypoxia, oxidative stress, and nutrient depletion can also induce autophagy and indirectly affect ctDNA. Targeting autophagy therapeutically with drugs that induce or inhibit autophagy such as chloroquine (CQ) or mechanistic target of rapamycin (mTOR) inhibitors can affect ctDNA concentrations. Although emerging mechanistic evidence suggests a potential relationship between autophagy and ctDNA dynamics, direct clinical studies validating this interaction remain lacking. Therefore, this review presents the autophagy–ctDNA relationship as a hypothetical and exploratory model that warrants further mechanistic and translational investigation in cancer development, therapeutic resistance, and clinical applications.

## 1. Introduction

Cancer is one of the most common causes of morbidity and mortality globally [1]. As global life expectancies rise coupled with environmental changes and lifestyle diseases, the incidence of cancer diagnoses will likely increase over time [2]. Although much progress has been made to target tumor-specific pathways and immune checkpoint blockades, early detection and tracking disease progression remain clinically challenging. Biopsy of tumor tissue is limited due to sampling error from tumor heterogeneity and inability to monitor disease in real time, and can be an invasive procedure for the patient [3]. In recent years, there has been increased interest in developing robust biomarkers that can be collected in a minimally invasive manner [4]. Liquid biopsy, the isolation and analysis of biomarkers from body fluids, has shown promising results, offering early detection and surveillance of disease [5].

Autophagy, a cellular “self-cleaning and recycling” process that removes damaged cellular components to maintain cell health, is a highly conserved catabolic process involving lysosomal degradation [6]. It has been shown to degrade damaged organelles and regulate the turnover of macromolecules [7]. Dysregulation of autophagy can lead to many diseases including cancer [8]. In early tumorigenesis, autophagy acts as a tumor suppressor gene by removing damaged proteins and DNA. As the tumor progresses, autophagy promotes tumorigenesis by allowing tumor cells to survive in harsh conditions such as hypoxia, nutrient deprivation, acidosis, and oxidative stress [9]. The regulation of autophagy involves many proteins, two of the most prevalent signaling pathways involved are AMPK and mTOR [10].

Cell-free DNA (cfDNA) is found throughout the bloodstream [11]. Circulating tumor DNA (ctDNA), as tumor-derived DNA fragments circulating in the bloodstream that can function as a molecular “fingerprint” of the tumor, is released by tumor cells and can be found within the greater pool of cfDNA [12]. ctDNA can harbor many tumor specific genetic and epigenetic changes that can be used to identify cancer within a patient [13]. Tracking changes in ctDNA can provide information on response to therapy and detection of minimal residual disease [14]. Liquid biopsy analyzing ctDNA offers several advantages over traditional biopsy methods including being less invasive and can provide information about tumor dynamics in real-time [15]. In this review, we explore the current understanding of autophagy and ctDNA in cancer biology and discuss emerging evidence suggesting a potential relationship between these processes. We present the proposed autophagy–ctDNA interaction as a hypothetical and exploratory model that warrants further mechanistic and translational investigation. Furthermore, we highlight how this conceptual framework may contribute to the development of clinically relevant liquid biopsy applications in precision oncology.

## 2. Molecular Biology of Autophagy in Cancer

**Figure 1:**
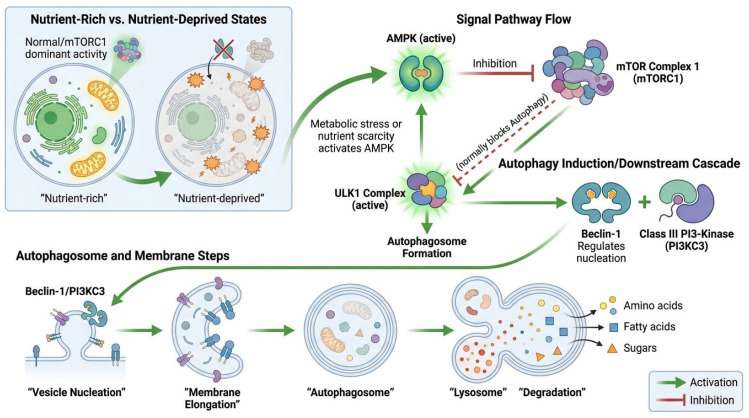
Molecular Biology of Autophagy in Cancer. In nutrient-abundant environments, mTOR complex 1 (mTORC1) remains operational and inhibits autophagy. Nutrient shortage or metabolic stress increases AMP-activated protein kinase (AMPK), which suppresses mTORC1 and triggers autophagy signaling. The activation of AMPK stimulates the ULK1 complex, which includes ULK1, ATG13, FIP200, and ATG101, so initiating the development of the autophagosome. The downstream cascade includes Beclin-1, which, in conjunction with class III phosphatidylinositol 3-kinase (PI3KC3), governs vesicle nucleation. This process is succeeded by membrane elongation and expansion, resulting in the creation of a double membrane autophagosome that encapsulates damaged organelles and macromolecules. Thereafter, the autophagosome merges with the lysosome to create an autolysosome, whereupon the cargo is decomposed into fundamental metabolites, including amino acids, fatty acids, and sugars, which are repurposed to sustain cellular metabolism.

Autophagy is a highly conserved cellular catabolic process involved in degrading and recycling damaged organelles, unfolded proteins, and macromolecules [16]. It plays a vital role in cellular homeostasis through lysosomal degradation. Two main upstream signals, AMPK, and mTOR regulate autophagy [17]. When nutrients are scarce or cells experience metabolic stress, AMPK becomes activated and negatively regulates mTOR complex 1 (mTORC1), which results in autophagy induction [18]. Then Unc-51-like kinase 1 (ULK1) complex becomes activated which induces autophagosome formation [19]. Finally, Beclin-1 helps in the regulation of vesicle nucleation through class III phosphatidylinositol 3-kinase (PI3KC3), and autophagic membrane elongation happens, resulting in degradation [20]. The pivotal function of the AMPK-mTOR-ULK1-Beclin-1 signaling pathway in modulating autophagy, highlighting its significance in sustaining cellular homeostasis and facilitating cancer cell adaptability to metabolic stress (Figure 1).

Autophagy can play both tumor-suppressive and tumor-promoting roles depending on the stage of tumor development [21]. It can help prevent tumor initiation early in tumorigenesis by suppressing genomic instability, oxidative stress, and the accumulation of damaged proteins/organelles [22]. For example, deletion or mutation of autophagy-related genes has been linked to tumor initiation. Autophagy can also help tumors grow later in tumor development by allowing cancer cells to survive during stressful conditions [23,24]. When autophagy is induced in tumor cells, it allows cancer cells to meet their metabolic requirements, become resistant to cell death, and better adapt to the tumor microenvironment.

Stress conditions such as hypoxia, nutrient deprivation, chemotherapy, and radiation are common in tumors [25]. Cancer cells can use autophagy to overcome these stresses and keep themselves alive. When tumors are low in nutrients, autophagy can recycle damaged cellular components to supply metabolites that can be used by the mitochondria to create energy and allow for continued biosynthesis [26]. Autophagy can also regulate signaling pathways involved in inflammation, immune responses, drug resistance, and radiotherapy resistance [27].

## 3. Circulating Tumor DNA: Origin and Clinical Applications

Circulating tumor DNA is part of cell-free DNA (DNA freely circulating in the bloodstream, which is released into the blood by tumor cells [28]. ctDNA contains tumor-specific genomic and epigenomic alterations and can be used as a biomarker in liquid biopsies to detect cancer or monitor disease progression [29].

### 3.1. Mechanisms of ctDNA Release

**Figure 2:**
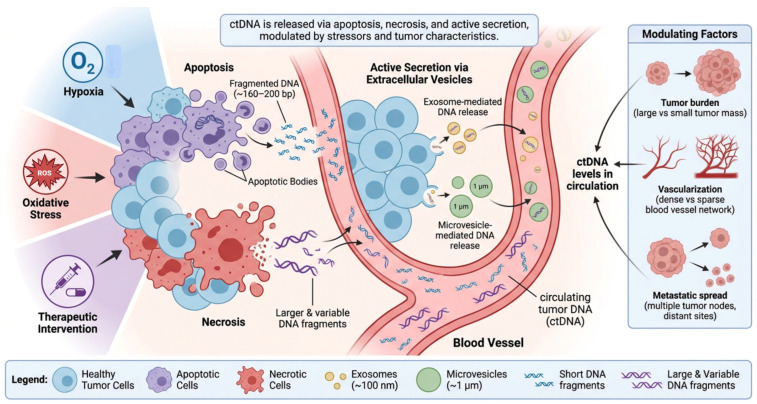
Mechanisms of circulating tumor DNA release. When tumor cells go through apoptosis, nucleosomes cut the DNA into very small pieces (about 160–200 bp). These pieces are then released into the bloodstream as apoptotic bodies. Necrosis, on the other hand, is often linked to fast tumor growth and poor blood flow, and it causes bigger and more diverse DNA fragments to be released. In addition to inactive release, tumor cells actively release DNA through exosomes (~100 nm) and microvesicles (~1 µm), which makes it easier for cells in the tumor microenvironment to talk to each other. Hypoxia, oxidative stress (ROS), and therapeutic approaches can all cause cells to shed ctDNA by killing tumor cells and changing the way membranes move. When ctDNA is released, it goes into the bloodstream and circulates as short and long DNA pieces that show genetic changes that are unique to the tumor. The amount of ctDNA in the blood is also affected by important tumor-related factors, such as the load (size and mass) of the tumor, its vascularization (number of blood vessels), and its metastatic spread (multiple tumor sites).

ctDNA released into circulation is thought to come from a variety of processes involved in tumor cell turnover. Passive release from apoptosis is thought to be the largest contributor to ctDNA [30]. During apoptosis, DNA becomes highly fragmented (~160–200 bp) due to nucleosomal cleavage [31]. Necrosis also releases DNA into circulation. Necrosis is common in tumors that experience rapid growth or inadequate blood supply, leading to heterogeneous fragments of varying size [32]. Cells can also actively release DNA from their cytoplasm through exosomes and microvesicles [33]. This form of DNA is thought to be used for intercellular communication between tumor cells. Cellular stress induced by hypoxia, oxidative stress, and treatment modalities have also been shown to increase shedding of ctDNA through cell death. The equilibrium between cell death and proliferation influences ctDNA abundance as well as fragment size [34]. Some biological processes can influence ctDNA levels such as tumor size, tumor vascularization, and metastasis (Figure 2).

### 3.2. ctDNA Characteristics and Detection Technologies

ctDNA is usually short fragment molecules of DNA in the circulating cell-free DNA pool. ctDNA represents a relatively rare fraction of total cfDNA and may be low abundance especially in early-stage cancers [35]. ctDNA carries tumor-specific aberrations such as point mutations, copy number aberrations, methylation and structural variants [36]. Methods have been established with sensitivity and specificity that allow rare variant detection. Methods used to detect ctDNA include ddPCR for quantification of known mutations and next generation sequencing for broad genomic profiling [37]. Amplicon-based targeted sequencing and hybrid capture targeted sequencing have been used to increase sensitivity to low abundance variants [38]. Novel technologies such as ultra-deep sequencing, methylation and fragmentomics-based assays are being developed. Factors before processing the sample also play a role in ctDNA detection [39].

### 3.3. Clinical Applications in Diagnosis, Prognosis, and Monitoring

ctDNA has shown clinical utility rapidly in many facets of cancer care. These include cancer diagnosis by non-invasively identifying mutations known to occur within a tumor when obtaining a tissue biopsy is difficult or impossible [40]. For cancer prognosis, increased ctDNA concentrations correlate with increased tumor burden, aggressive disease, and poor outcome [41]. Analysis of ctDNA has been useful for monitoring treatment response as the change in ctDNA detected in blood can closely follow tumor changes often before they are detectable on imaging studies [42]. It has also been used to assess minimal residual disease after treatment has been completed allowing earlier identification of relapse. Resistance mutations have also been detected with ctDNA to help guide therapy and allow personalized medicine [43]. As liquid biopsy technology continues to advance, ctDNA will continue to be used for precision medicine in cancer diagnosis.

## 4. Mechanistic Interplay Between Autophagy and ctDNA

Autophagy has emerged as a regulator of ctDNA levels. Autophagy plays numerous roles in tumor cell survival, death, and genomic stability. Because of these functions, autophagy can regulate the amount of ctDNA shed by tumor cells under normal circumstances and conditions of stress such as therapy and microenvironmental stress.

### 4.1. Autophagy-Regulated Cell Death and DNA Release

Autophagy has been shown to determine tumor cell fate directly affecting ctDNA release [44]. Under basal or moderate stress conditions, autophagy prevents cell death promoting cell survival. Therefore, when autophagy activity is increased, apoptotic and necrotic cell death are diminished slowing ctDNA release into the bloodstream [45]. However, when tumor cells are exposed to high amounts of stress or pharmacological inhibition of autophagy occurs, apoptosis, necrosis or autophagy-dependent cell death occurs leading to DNA shedding [46]. More specifically, autophagy has been shown to delay apoptosis by interfering with apoptotic signaling. This interference occurs due to autophagy reducing mitochondrial damage and buildup of reactive oxygen species. Furthermore, when autophagic flux is blocked or inhibited, there is increased cellular stress leading to DNA release [47]. In line with these studies, inhibition of autophagy using chloroquine or activation of autophagy using mTOR inhibitors change ctDNA levels in response to therapy.

### 4.2. Genomic Instability and DNA Fragmentation

**Figure 3:**
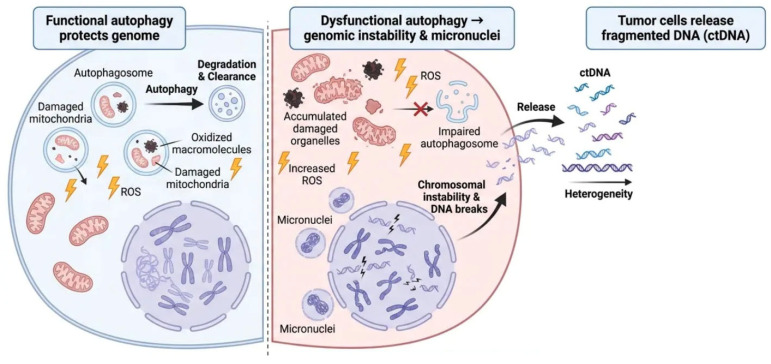
Mechanistic interplay between autophagy and ctDNA in genomic instability and DNA fragmentation. Under normal settings, functioning autophagy maintains genomic stability by promoting the degradation and removal of damaged mitochondria, oxidized macromolecules, and excess ROS. Autophagy, via autophagosome production and lysosomal degradation, inhibits the accumulation of cellular damage, thereby mitigating DNA damage and preserving chromosomal integrity. Dysfunctional or defective autophagy results in the accumulation of damaged organelles and raised amounts of ROS, leading to increased oxidative stress. This induces chromosomal instability, DNA double-strand breaks, and micronuclei production, which are essential characteristics of genomic instability. Impaired autophagosome formation exacerbates cellular injury by altering typical degradation processes. Consequently, tumor cells with impaired autophagy release elevated levels of fragmented DNA into the extracellular environment, thereby contributing to ctDNA. The ctDNA fragments display variability in size and sequence, indicating genetic changes present in tumor cells. Cross indicated no interaction or inhibition.

Autophagy is intimately involved in maintenance of genomic stability, and deregulation of autophagy has been shown to promote genomic instability, which is observed in cancer [48]. Autophagy promotes degradation of damaged mitochondria and oxidized macromolecules that otherwise cause excess ROS and subsequent DNA damage [49]. Dysfunction in autophagy leads to accumulation of damaged organelles, oxidative stress, DNA double-strand breaks, chromosomal instability, and micronucleus formation [50]. These defects may allow cells to release degraded fragments of DNA into the extracellular space, driving increases in ctDNA levels [51]. Dysfunctional autophagy may also influence DNA degradation pathways to produce ctDNA fragments of heterogeneous lengths, altering fragmentomics profiles [52]. Quality and fragmentation patterns of ctDNA released by tumors may therefore provide insight into autophagy activity within tumors and could be used alongside ctDNA mutation profiles to monitor tumor evolution, genomic instability and progression of disease [39]. Figure 3 depicts the function of autophagy in preserving genomic integrity and its influence on the release of circulating tumor DNA. Autophagy malfunction is a significant catalyst for DNA fragmentation and ctDNA release, connecting intracellular stress responses to extracellular biomarker fluctuations in cancer [53].

### 4.3. Impact of Tumor Microenvironment (Hypoxia, ROS)

Another source of regulation for the autophagy–ctDNA axis lies within the TME. Stressors in the TME such as hypoxia, oxidative stress, and nutrient depletion can regulate both autophagy and ctDNA release [54]. Hypoxic conditions upregulate hypoxia-inducible factors that subsequently induce autophagy, allowing tumor cells to adapt to decreased oxygen availability [55]. Likewise, increased ROS levels can induce autophagy to prevent oxidative damage. Decreased cell death through these mechanisms may blunt ctDNA release in experimental settings [56]. However, overwhelming stress responses can override autophagy mediated adaptation, resulting in ROS accumulation and prolonged hypoxia-inducible factor activation, which ultimately leads to cell death and ctDNA release [57]. Therapeutic stressors such as chemotherapy and radiotherapy can also increase oxidative stress and inhibit autophagy.

### 4.4. Secretory Autophagy and Active ctDNA Release Through Extracellular Vesicles

Besides passive release via apoptosis and necrosis, ctDNA may potentially be actively secreted through autophagy-related extracellular vesicle pathways. Recent data indicates that secretory autophagy, a non-canonical variant of autophagy, facilitates the extracellular release of intracellular materials, including as nucleic acids, proteins, and damaged cellular components [58]. In contrast to degradative autophagy, which involves the fusion of autophagosomes with lysosomes for the breakdown of cargo, secretory autophagy drives autophagic vesicles to the plasma membrane for extracellular secretion [59].

Proteins associated with autophagy, such as LC3, ATG proteins, and RAB family GTPases like RAB27A and RAB8A, are crucial for vesicle trafficking, docking, and secretion [60]. LC3-associated secretory pathways enable the encapsulation of cytoplasmic substances into extracellular vesicles, such as exosomes and microvesicles, which may transport tumor-derived DNA fragments into the bloodstream [61]. In settings of hypoxia, oxidative stress, inflammation, or therapy-induced cellular damage, cancer cells may enhance secretory autophagy activity as a survival adaptation [62].

Extracellular vesicles can facilitate intercellular communication within the tumor microenvironment and may play a role in tumor growth, immunological regulation, and metastatic spread [63]. Significantly, DNA-containing exosomes generated via autophagy-related mechanisms may serve as an active source of ctDNA independent of cellular apoptosis [64]. This method offers a physiologically credible rationale for the detection of ctDNA in tumors exhibiting minimal apoptosis or necrosis. While the exact molecular mechanisms governing autophagy-dependent ctDNA secretion are not fully elucidated, incorporating secretory autophagy into the autophagy–ctDNA framework enhances the mechanistic comprehension of ctDNA biology and underscores novel avenues for translational liquid biopsy research in oncology.

### 4.5. Autophagy Interactions with Regulated Cell Death Pathways and Their Impact on ctDNA Release

Alongside apoptosis and necrosis, various kinds of controlled cell death, such as ferroptosis, pyroptosis, necroptosis, and parthanatos, are becoming acknowledged as significant factors in cancer progression and the release of ctDNA [65]. These mechanisms demonstrate substantial mechanistic connections with autophagy and may produce unique DNA fragmentation patterns that affect ctDNA composition and the interpretation of liquid biopsies.

Ferroptosis is an iron-dependent mechanism of controlled cell death marked by severe lipid peroxidation and oxidative damage to membranes. Autophagy facilitates ferroptosis via the selective breakdown of ferritin, termed ferritinophagy, hence augmenting intracellular iron levels and enhancing the production of reactive oxygen species [66]. Augmented ferroptotic stress may cause damage to tumor cell membranes and DNA fragmentation, potentially facilitating the release of circulating ctDNA [67].

Pyroptosis is an inflammatory variant of programmed cell death driven by inflammasome activation and the development of gasdermin-dependent membrane pores [68]. Autophagy can inhibit inflammasome activation by eliminating damaged mitochondria and diminishing inflammatory signals. Conversely, compromised autophagy may exacerbate pyroptotic cell death and elevate extracellular DNA release via membrane rupture and inflammatory tissue damage [69].

Necroptosis, governed by receptor-interacting protein kinases RIPK1 and RIPK3, as well as mixed lineage kinase domain-like protein (MLKL), also crosses with autophagy pathways [70]. Dysregulated autophagy may amplify necroptotic signals and facilitate extensive DNA release resulting from plasma membrane breakdown [71]. Parthanatos, a PARP1-dependent cell death pathway linked to significant DNA damage and mitochondrial malfunction, may be affected by oxidative stress and impaired autophagic clearance systems [72]. The controlled cell death mechanisms collectively enhance the mechanistic understanding of the suggested autophagy–ctDNA link, indicating that different types of tumor cell death may yield varied ctDNA release kinetics and fragmentation patterns in cancer patients.

## 5. Role of the cGAS–STING Pathway in the Autophagy–ctDNA Relationship

**Figure 4:**
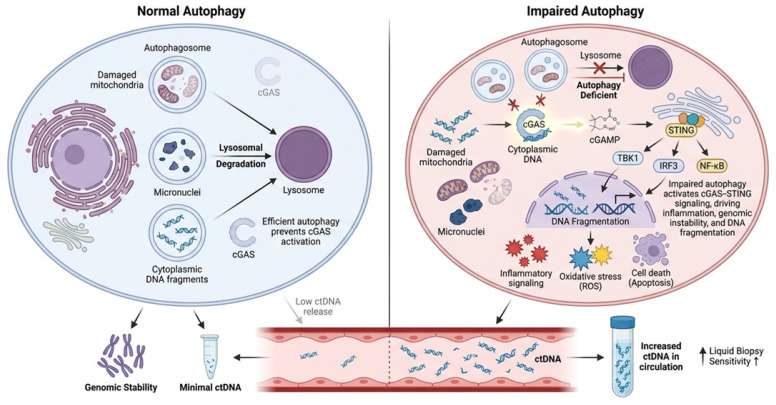
Role of the cGAS-STING Pathway in the Autophagy–ctDNA. Under normal autophagic conditions, impaired mitochondria, micronuclei, and cytoplasmic DNA fragments are effectively eliminated by lysosomal degradation, therefore averting excessive activation of the cGAS-STING pathway and preserving genomic integrity. As a result, the release of ctDNA into circulation stays limited. Conversely, compromised autophagy hinders autophagosome-lysosome destruction, resulting in the buildup of cytoplasmic DNA, damaged organelles, and micronuclei. Cytoplasmic DNA triggers cGAS, leading to the production of cyclic GMP-AMP (cGAMP) and the stimulation of STING signaling. Activated STING triggers downstream pathways involving TBK1, IRF3, and NF-κB, hence enhancing inflammatory signaling, oxidative stress, apoptosis, DNA fragmentation, and genomic instability. These activities augment the release of tumor-derived ctDNA into circulation, potentially enhancing the sensitivity of liquid biopsies and indicating persistent tumor stress and genetic damage. Cross indicated no interaction or inhibition.

The cyclic GMP-AMP synthase (cGAS)-stimulator of interferon genes (STING) pathway is widely acknowledged as a pivotal molecular connection among autophagy, genomic instability, inflammation, and circulating tumor DNA (ctDNA) dynamics in cancer [73]. cGAS operates as a cytoplasmic DNA sensor that identifies double-stranded DNA originating from compromised nuclei, micronuclei, mitochondria, or pathogenic invaders [74]. Upon binding to cytoplasmic DNA, cGAS synthesizes cyclic GMP–AMP (cGAMP), activating STING and subsequently initiating downstream inflammatory signaling pathways, including TBK1, IRF3, and NF-κB, leading to the production of type I interferon and pro-inflammatory cytokines [75]. Figure 4 illustrates the mechanistic relationship between autophagy, cGAS-STING signaling, genomic instability, and ctDNA release in cancer cells.

Autophagy is essential in modulating this route by eliminating damaged mitochondria, cytoplasmic chromatin fragments, and micronuclei via lysosomal degradation. Functional autophagy thus inhibits the excessive buildup of cytoplasmic DNA and restricts the inappropriate activation of cGAS-STING signaling [76]. Conversely, dysfunctional or deficient autophagy facilitates the retention of cytoplasmic DNA and micronuclei, hence augmenting cGAS-STING activation and exacerbating chronic inflammation, oxidative stress, and genomic instability [77]. These events may expedite tumor development and modify tumor cell death mechanisms.

The stimulation of the cGAS-STING pathway may significantly affect the kinetics of ctDNA release [78]. Chronic inflammatory signaling and genomic instability can elevate apoptosis, necrosis, and stress-induced cell death, thereby augmenting the release of fragmented tumor DNA into the circulatory system [79]. Moreover, DNA damage linked to cGAS-STING activation may result in diverse ctDNA fragment patterns indicative of persistent chromosomal instability [80].

Recent findings indicate bidirectional connections between autophagy and STING signaling, wherein STING activation can stimulate autophagy, while autophagy adversely modulates STING activity via degradation pathways [81]. While direct experimental evidence connecting cGAS-STING activity with ctDNA kinetics is scarce, this pathway offers a biologically plausible mechanistic framework that underpins the suggested autophagy–ctDNA relationship, necessitating further mechanistic and translational exploration in cancer research.

## 6. Autophagy, ctDNA, and Therapeutic Resistance

**Figure 5:**
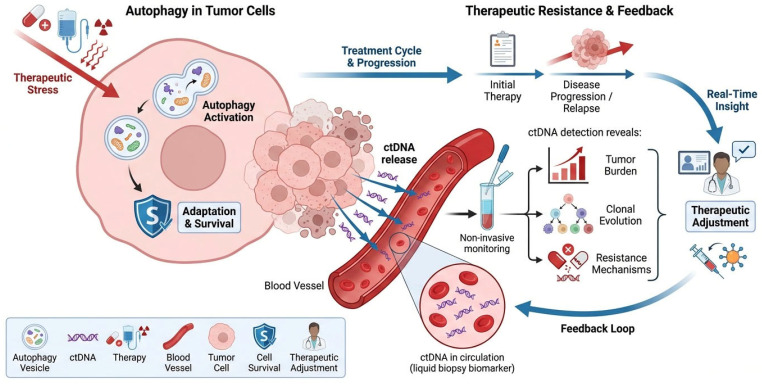
Autophagy, ctDNA, and therapeutic resistance. Tumor cells use autophagy as an adaptive survival strategy while under therapeutic stress, such as chemotherapy, radiation, or targeted therapy. By facilitating the recycling of intracellular components, autophagy helps tumor cells tolerate oxidative and metabolic stress, which increases cell survival and treatment resistance. A portion of the tumor cells die as the treatment goes on, releasing fragmented DNA into the bloodstream as ctDNA. As a non-invasive liquid biopsy biomarker, this ctDNA represents molecular changes and tumor load. Real-time insights into tumor dynamics, such as shifts in tumor burden, clonal evolution, and the formation of resistance mechanisms, are provided by ctDNA analysis through serial monitoring. A feedback loop in which clinical decision-making is informed by ctDNA-based detection, allowing for therapeutic modification and treatment strategy optimization. Persistent autophagy-driven survival can reinforce resistance pathways and contribute to disease progression or relapses even in the face of initial treatment responses.

Autophagy and ctDNA are closely linked to therapeutic resistance in cancer. Autophagy promotes tumor adaptation and survival under treatment stress, while ctDNA provides a dynamic, non-invasive biomarker reflecting tumor burden, clonal evolution, and resistance mechanisms, offering valuable insights into treatment response and disease progression in real time (Figure 5).

### 6.1. Role in Chemoresistance and Tumor Adaptation

Autophagy has been shown to mediate chemoresistance and tumor adaptation to chemotherapy. Apoptosis-inducing agents used in cancer treatment include cytotoxic chemotherapy and radiotherapy induce metabolic stress, DNA damage and oxidative stress, which are mediated by activation of AMPK and inhibition of mTOR leading to autophagy [82]. Autophagy induction during therapy helps tumor cells recycle intracellular stores to replenish nutrients and prevent apoptotic cell death. Autophagy has been shown to promote resistance to cytotoxic chemotherapy including platinum compounds and temozolomide in ovarian cancer and glioblastoma, respectively [83]. Furthermore, autophagy has also been shown to maintain stem-like cancer cell survival and induce epithelial–mesenchymal transition. However, autophagy induction has also been shown to induce autophagy dependent cell death in some contexts. Pharmacological inhibitors of autophagy like chloroquine or hydroxychloroquine can be used to inhibit autophagy to sensitize tumors to therapy [84].

### 6.2. ctDNA as a Biomarker of Treatment Response

ctDNA can be utilized as a biomarker of response to treatment and development of resistance [85]. Clinical evidence has shown that ctDNA levels can be correlated with tumor burden, whereby clearance of ctDNA during treatment indicates a response to therapy and persistent ctDNA can indicate resistance to therapy or progressive disease [86]. ctDNA can allow us to detect development of resistance mechanisms such as DNA repair mutations or activated oncogenic signaling mutations before failure is detected on clinical or radiographic assessment [87]. ctDNA assays also have the benefit of characterizing tumor heterogeneity and clonal selection across different tumor sites including both the primary and metastatic lesions [88]. ctDNA levels can transiently rise after administration of therapy due to therapy induced cell death. This phenomenon has been referred to as ctDNA “flare”. Integration of ctDNA profiling with autophagy modulation could allow us to monitor tumor response dynamics to inform precision treatment decisions [89].

### 6.3. Evidence from Preclinical and Clinical Studies

Autophagy inhibition has been shown to sensitize to chemotherapy and increase tumor cell death in preclinical models. This has been shown along with increased release of ctDNA [90]. Ovarian cancer and glioblastoma treated with autophagy inhibitors along standard of care therapies showed sensitization to treatment in preclinical models. Liquid biopsy has been used clinically as validated ctDNA analysis to monitor treatment response, detect minimal residual disease, and discover resistance mutations [91]. This has been utilized in various cancers. Studies looking at autophagy inhibition or stimulation and ctDNA in patients are limited [92]. Clinical trials underway with agents modulating autophagy along with liquid biopsy strategy will likely reveal answers mechanistically and translationally.

Numerous clinical trials have examined autophagy inhibitors, including CQ and HCQ, in conjunction with chemotherapy, endocrine therapy, targeted therapy, or immunotherapy in cancer patients (Table 1). The phase I/II trial NCT03774472 assessed the efficacy of hydroxychloroquine in conjunction with palbociclib and letrozole for breast cancer, incorporating exploratory analysis of blood-based DNA and RNA biomarkers related to autophagy and tumor response [93]. A concurrent phase Ib/II trial, NCT05953350, is examining hydroxychloroquine in conjunction with CDK4/6 inhibitors in solid tumors to evaluate safety, effectiveness, and translational biomarker responses [94]. Despite the limited availability of direct ctDNA-focused outcomes, these findings illustrate a growing incorporation of liquid biopsy-related indicators into treatment trials targeting autophagy. Recent evidence indicates that ctDNA monitoring may offer real-time insights into tumor stress produced by treatment, therapeutic response, and the evolution of resistance during autophagy modulation [93]. These translational results endorse the potential incorporation of ctDNA profiling into autophagy-oriented precision cancer methods.

## 7. Therapeutic Targeting of the Autophagy–ctDNA Axis

Targeting the autophagy–ctDNA axis therapeutically may help improve cancer therapy response and biomarker monitoring. Altering autophagy can impact both tumor cell survival/death and ctDNA release kinetics [96]. Pharmacologic interventions may therefore be employed to sensitize tumors to treatment and monitor response to treatment through liquid biopsies.

### 7.1. Autophagy Modulators (Chloroquine, Hydroxychloroquine, mTOR Inhibitors)

**Figure 6:**
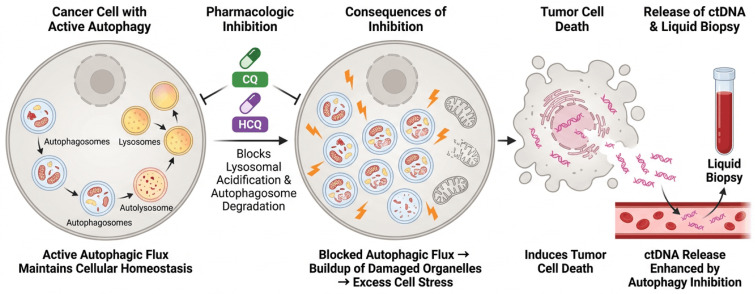
Targeting autophagy in cancer therapy and effects on tumor cell death and ctDNA release. Autophagosomes and lysosomes combine to generate autolysosomes in cancer cells that have active autophagy. This allows for the effective breakdown of damaged organelles and the continual autophagic flux that maintains cellular homeostasis. By limiting autophagosome disintegration and lysosomal acidification, pharmacological drugs like HCQ and CQ restrict autophagy. Damaged organelles build up, cellular stress increases, and reactive oxygen species levels rise because of this disturbance, which also impairs autophagic flow. As a result, autophagy inhibition encourages tumor cell death, mostly by necrosis and apoptosis. Tumor cell disintegration causes fragmented DNA to be released into the extracellular space, which raises the amount of ctDNA in the blood. By making tumor-derived genetic material more detectable using liquid biopsy, this increased ctDNA shedding makes it possible to monitor tumor dynamics and treatment response non-invasively.

Targeting autophagy directly with pharmacologic agents has shown great potential as a cancer therapy. CQ and HCQ are among the most used autophagy inhibitors [97]. By preventing lysosomal acidification and disrupting autophagosome degradation, they block autophagic flux, causing buildup of damaged organelles and excess cell stress that ultimately induces tumor cell death (Figure 6). Drugs have been studied both preclinically and clinically in models both alone and in combination with chemotherapeutics or other targeted agents to overcome resistance.

Alternatively, autophagy can be modulated upstream with drugs that inhibit mTOR, a major negative regulator of autophagy [98]. Treatment with inhibitors of mTOR such as rapamycin causes activation of autophagy by disinhibiting ULK1 complex [99]. While activation of autophagy can help tumors become more resistant to cell death, depending on tumor characteristics/context and combination with other therapies tumor cell death can be promoted. Due to autophagy’s dual role in cancer, either inhibiting or activating it can be used therapeutically, depending on the situation. It is also important to note that modulation of autophagy affects release of ctDNA by changing mechanisms of tumor cell death, and thus agents that target autophagy may indirectly alter ctDNA levels and aid in liquid biopsy readouts while on treatment [100].

### 7.2. Combination Therapies and Precision Medicine Approaches

Autophagy modulators can also be combined with other treatment modalities to enhance therapeutic outcomes or overcome resistance. For example, autophagy inhibitors like chloroquine are often used in combination with chemotherapy, radiation therapy, or targeted therapies to potentiate cell death by preventing autophagy-mediated cell survival [101]. Conversely, strategies that induce autophagy may be combined with therapies that selectively kill cells undergoing autophagy or boost immune responses against tumor cells [101]. Precision medicine strategies could involve using molecular profiling techniques, such as ctDNA analysis, to identify mutations specific to a patient’s tumor and tailor treatment accordingly [102]. Monitoring ctDNA levels and changes during treatment can provide information about treatment efficacy and emerging resistance [103]. Combining autophagy-targeting approaches with emerging therapies, such as nanoparticle-based drug delivery systems or immunotherapy, may also enhance specificity and efficacy against tumor cells [104].

### 7.3. Potential to Enhance ctDNA-Based Monitoring

Autophagy modulation may also serve as a tool to improve ctDNA-based monitoring. Autophagy inhibitors can enhance tumor cell death and the kinetics of ctDNA release, increasing ctDNA shedding [105]. Induction of cytoprotective autophagy could dampen ctDNA release in response to therapy [106]. Autophagy inhibitors could therefore aid ctDNA-based detection of treatment response. Combined use of autophagy-targeting agents with ctDNA analysis could increase the sensitivity of liquid biopsy to detect treatment response early and may reveal a more accurate picture of tumor evolution and therapy resistance in cancer [107].

## 8. Translational and Clinical Perspectives

One possibility of translating autophagy markers into the clinical setting is in combination with ctDNA detection. Both modalities present tumor specific information about the functioning of cells within tumors, where autophagy markers can be used to describe the physiological processes occurring within tumor cells, such as LC3-II and p62/SQSTM1 levels indicating autophagic flux, while ctDNA provides genetic information about the tumor in real time [108]. Monitoring both together could lead to greater understanding of how these processes correlate with genomic events in the tumor. Another use case includes correlating autophagy biomarkers with ctDNA to better understand the context of treatment response when therapeutic agents are being used that have cytoprotective or cytotoxic effects mediated by autophagy [109].

The effective clinical use of the proposed autophagy–ctDNA system necessitates rigorous analytical validation and standardized biomarker integration methodologies. Autophagy-related biomarkers, such as serum LC3-II, p62/SQSTM1, Beclin-1, and extracellular vesicle-associated proteins, may offer supplementary insights into tumor stress adaptation and therapeutic response when integrated with ctDNA profiling [110]. Nonetheless, inconsistencies in autophagic flux evaluation, specimen handling, and test repeatability continue to pose significant challenges. Standardized pre-analytical procedures, encompassing blood collection, plasma isolation, and biomarker storage, are crucial to reduce technical variability and enhance assay sensitivity.

The incorporation of autophagy biomarkers with ctDNA tests necessitates thorough clinical validation and regulatory qualification to confirm analytical specificity, repeatability, and clinical value [111]. Regulatory agencies are placing greater emphasis on the harmonization of biomarkers, the creation of companion diagnostics, and the establishment of standardized validation frameworks for precision oncology applications.

Co-clinical trial designs offer significant opportunity for the integration of serial ctDNA monitoring with the pharmacodynamic evaluation of autophagy markers during therapy with autophagy modulators, like chloroquine, hydroxychloroquine, or mTOR inhibitors [112]. These studies may enhance patient classification, elucidate early resistance mechanisms, and optimize therapy monitoring measures. Future multi-omics and long-term clinical investigations will be crucial for evaluating the translational potential of the hypothesized autophagy–ctDNA concept in cancer treatment.

While exciting, there are hurdles that must be overcome before clinical adoption of this combination can occur. As mentioned before, standardized utilization and reporting of autophagy markers are not prevalent yet. Furthermore, most of the markers mentioned require tissue samples, and their expression can be dynamic as autophagy is constantly turned on and off in cells. With ctDNA, although a liquid biopsy, its abundance is often low in early disease, and pre-analytical variables can affect results. Longitudinal clinical trials validating the usage of these tests together will be necessary.

## 9. Limitations and Future Directions

Although promising, there are several limitations and gaps in our current knowledge regarding the autophagy–ctDNA axis. The connection between autophagy and ctDNA has primarily been established through indirect evidence from preclinical models, with limited validation in patient samples. The dual role of autophagy in cancer, acting as both tumor suppressor and promoter depending on the context, may confound its relationship with ctDNA release [113]. Additionally, inconsistent measurements of autophagy markers, such as discrepancies in assessing autophagic flux, as well as variations in ctDNA detection sensitivity, have hindered robust correlations between the two processes [114].

Clinical translation of the autophagy–ctDNA axis necessitates well-designed longitudinal studies that assess both autophagy activity and ctDNA levels during disease progression and treatment response. Incorporating multi-omics analyses, such as genomics, transcriptomics, proteomics, and metabolomics, could provide a more comprehensive understanding of the molecular mechanisms driving this relationship and aid in the identification of potential biomarkers. Ultra-sensitive sequencing, single-cell analyses, and advanced imaging modalities represent emerging techniques that could further elucidate the intricacies of the autophagy–ctDNA axis [115]. These approaches hold promises for advancing our understanding and translating these findings into clinically relevant applications for cancer diagnosis, monitoring, and treatment.

Present evidence directly associating autophagy with ctDNA is scarce, primarily consisting of indirect mechanistic insights and preclinical investigations. Variability in autophagy evaluation, sensitivity of ctDNA detection, and tumor heterogeneity further obfuscate interpretation [116]. Future research should employ longitudinal experimental and clinical designs that integrate autophagy markers with ctDNA profiling through multi-omics techniques, encompassing genomics, transcriptomics, proteomics, and metabolomics. Innovative technologies like ultra-sensitive sequencing and single-cell analysis may elucidate this relationship further. Consequently, the suggested autophagy–ctDNA connection should presently be regarded as a theoretical and exploratory framework that necessitates additional mechanistic and translational research in cancer biology and precision oncology.

## 10. Conclusions

The autophagy–circulating tumor DNA axis constitutes a unique and dynamic interface in cancer biology, connecting internal stress adaptation with the release of extracellular biomarkers. Autophagy has a dual function in tumor growth, influencing cell survival, genomic stability, and therapeutic response, whilst ctDNA acts as a minimally invasive biomarker indicative of tumor burden and molecular evolution. The interaction between these processes’ underscores how the manipulation of autophagy might affect ctDNA release, composition, and clinical interpretability. Clinically, the integration of autophagy markers with ctDNA profiling presents a promising approach to enhance therapy monitoring, identify early resistance, and optimize patient classification in precision oncology. Nonetheless, additional validation is necessary to establish consistent methodologies and strong clinical connections. Future advancements in multi-omics technologies and liquid biopsy platforms are anticipated to expedite the integration of this axis into clinical practice, hence improving early diagnosis, real-time monitoring, and individualized therapy strategies in cancer management.

## Figures and Tables

**Table 1:** Clinical trial evidence supporting autophagy modulation and liquid biopsy applications.

Clinical Trial (NCT)	Cancer Type	Autophagy Modulator	Combination Therapy	Phase	Biomarker/Liquid Biopsy Endpoint	Key Focus	Ref.
NCT03774472	Breast cancer	Hydroxychloroquine (HCQ)	Palbociclib + Letrozole	Phase I/II	Blood-based DNA/RNA biomarkers	Autophagy, senescence, treatment response	[93]
NCT05953350	Solid tumors	Hydroxychloroquine (HCQ)	CDK4/6 inhibitors	Phase Ib/II	Exploratory translational biomarkers	Safety, efficacy, autophagy inhibition	[94,95]
PALAVY Study	Stage II–III breast cancer	Hydroxychloroquine sulfate	±Palbociclib/Avelumab	Phase II	Disseminated tumor cell monitoring	Dormancy and resistance mechanisms	[93]

## Data Availability

No new data were created or analyzed in this study.
